# 2135: A novel treatment delivery of acceptance and commitment therapy for chronic pain in an integrated primary care setting

**DOI:** 10.1017/cts.2017.245

**Published:** 2018-05-10

**Authors:** Kathryn E. Kanzler, Patricia Robinson, Mariana Munante, Donald McGeary, Jennifer Potter, Bria MacIntyre, Eliot Lopez, Willie Hale, James Mintz, Dawn Velligan

**Affiliations:** University of Texas Health Science Center San Antonio, San Antonio, TX, USA

## Abstract

OBJECTIVES/SPECIFIC AIMS: This study seeks to test the feasibility and effectiveness of a brief acceptance and commitment therapy (ACT) treatment for chronic pain patients in a primary care clinic METHODS/STUDY POPULATION: Primary care patients aged 18 years and older with at least 1 pain condition for 12 weeks or more in duration will be recruited. Patients will be randomized into (a) ACT intervention or (b) control group. Participants in the ACT arm will attend 1 individual visit with an integrated behavioral health provider, followed by 3 weekly ACT classes and a booster class 2 months later. Control group will receive enhanced primary care that includes patient education handouts informed by cognitive behavioral science. Data analysis will include 1-way analysis of covariance (ANCOVA), multiple regression with bootstrapping. RESULTS/ANTICIPATED RESULTS: The overall hypothesis is that brief ACT treatment reduces physical disability, improves functioning, and reduces medication misuse in chronic pain patients when delivered by an integrated behavioral health provider in primary care. In addition, it is anticipated that improvements in patient functioning will be mediated by patient change in pain acceptance and patient engagement in value-consistent behaviors. DISCUSSION/SIGNIFICANCE OF IMPACT: This pilot study will establish preliminary data about the effectiveness of addressing chronic pain in a generalizable integrated primary care setting. Data will help support a larger trial in the future. Findings have potential to transform the way chronic pain is currently managed in primary care settings, with results that could decrease disability and improve functioning among patients suffering from chronic pain.

